# Effect of radiotherapy after breast-conserving surgery in older patients with early breast cancer and breast ductal carcinoma *in situ*: a meta-analysis

**DOI:** 10.18632/oncotarget.15998

**Published:** 2017-03-08

**Authors:** Xuan-zhang Huang, You Chen, Wen-jun Chen, Xi Zhang, Cong-cong Wu, Chao-ying Zhang, Shuang-shuang Sun, Jian Wu

**Affiliations:** ^1^ Department of Chemotherapy and Radiotherapy, The Second Affiliated Hospital and Yuying Children's Hospital of Wenzhou Medical University, Wenzhou City 325027, P.R. China; ^2^ The Wenzhou Dental Hospital, Wenzhou City 325027, P.R. China

**Keywords:** radiotherapy, breast-conserving surgery, older, early breast cancer, breast ductal carcinoma *in situ*

## Abstract

**Background:**

There are no consistent agreements on whether radiotherapy after breast-conserving surgery (BCS) could provide local control and survival benefit for older patients with early breast cancer or breast ductal carcinoma *in situ* (DCIS). The present study aimed to evaluate the efficacy of radiotherapy after BCS in older patients with early breast cancer or DCIS.

**Results:**

Radiotherapy could reduce the risk of local relapse in older patients with early breast cancer. The 5-year AR of local relapse was 2.2% and 6.2% for radiotherapy and non-radiotherapy group, respectively, with low 5-year ARD of 4.0% and high NNT of 25. The 10-year AR of local relapse was 5.3% and 10.5% for radiotherapy and non-radiotherapy group, respectively, with the 10-year ARD of 5.2% and NNT of 20. However, radiotherapy could not improve survival benefits, including overall survival, cancer-specific survival, breast-cancer-specific survival, and distant relapse. Moreover, radiotherapy could reduce the risk of ipsilateral breast events in older patients with DCIS.

**Materials and Methods:**

PubMed and Embase database were searched for relevant studies. Hazard ratios (HRs), risk ratios (RRs), absolute risk (AR), absolute risk difference (ARD), and number needed to treat (NNT) were used as effect measures to evaluate the efficacy of radiotherapy in older patients.

**Conclusions:**

Our study indicates that radiotherapy could slightly reduce the risk of local relapse in older patients with favorable early breast cancer. However, radiotherapy cannot translate into significant survival benefits.

## INTRODUCTION

Breast cancer is a global health issue in women worldwide, and the incidence of breast cancer has risen in women with older age [1–3]. Although postoperative breast radiotherapy is the standard treatment following breast-conserving surgery (BCS) in early breast cancer, the treatment recommendations for postoperative radiotherapy do not consider age as a treatment factor and there is a lack of high-level evidence for the efficacy of postoperative radiotherapy after BCS in older patients with early breast cancer [4]. Moreover, it is inappropriate to extrapolate the results of studies in younger patients to older patients, because older patients may have high comorbidity and thus are frequently excluded from studies [5, 6].

Older patients may have less aggressive tumor biology and the postmenopausal status contributes to a lower risk of tumor relapse [7, 8]. Besides, the high risks of comorbidity in older patients are associated with increased complications and decreased tolerability of treatment and short life expectancy. Several studies have showed that the use of postoperative radiotherapy after BCS appears to have declined in older patients with early breast cancer [9, 10]. Thus, it is difficult and important to explore the efficacy of postoperative radiotherapy in older patients with early breast cancer [11, 12]. However, it remains controversial whether an omission of radiation therapy can be considered for older patients with early breast cancer.

Thus, the purpose of this study was to evaluate the efficacy of postoperative radiotherapy after BCS in older patients with early breast cancer and whether the efficacy of postoperative radiotherapy differed according to factors such as age and tumor characteristics. Moreover, we also assessed the efficacy of postoperative radiotherapy for older patients with breast ductal carcinoma *in situ* (DCIS).

## RESULTS

### Baseline characteristics of the included studies

**Figure 1:**
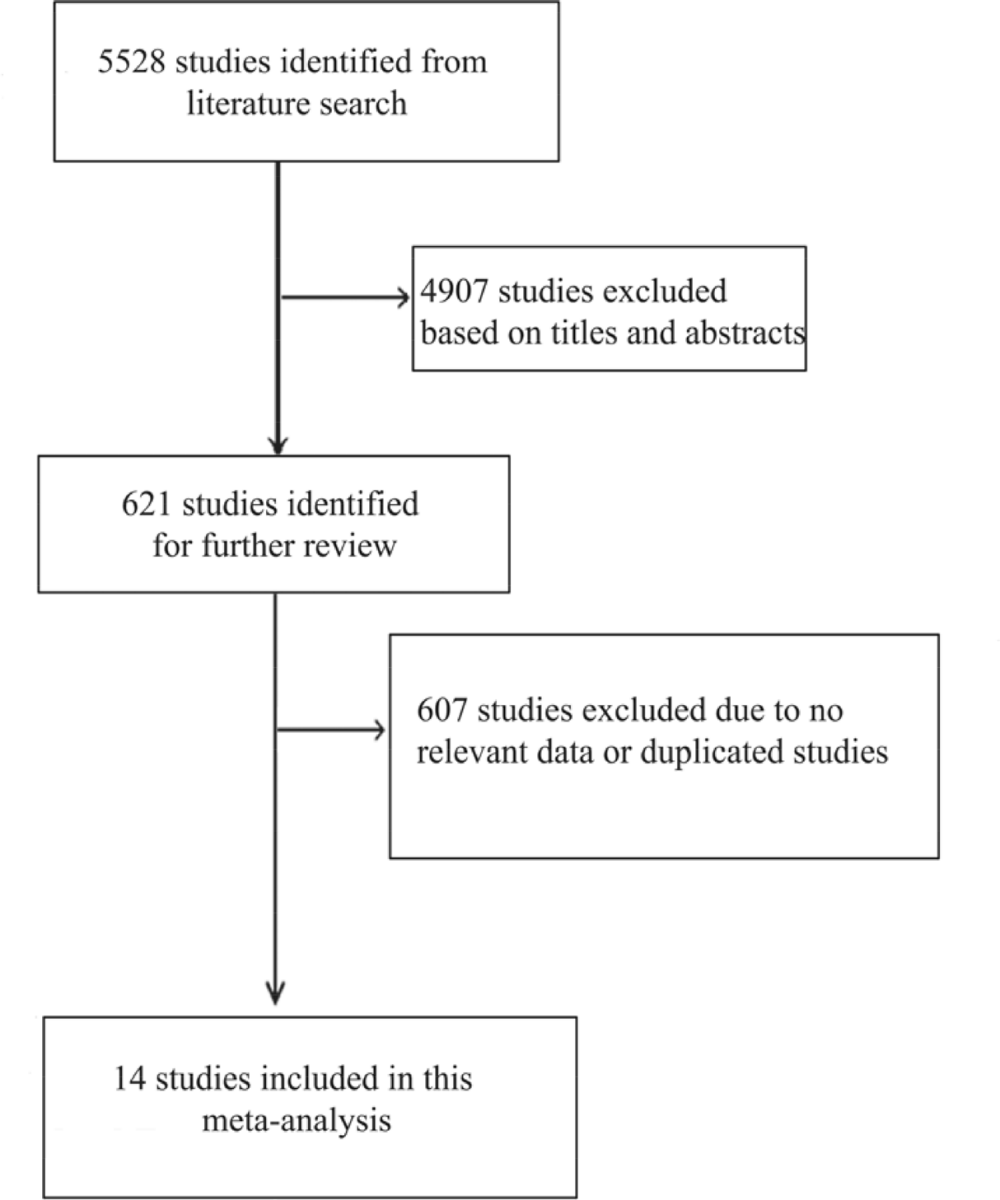
Flow diagram showing the literature search and study selection

A total of 5528 relevant studies were identified from literature search, of which 4907 were excluded after screening the titles and abstracts. The remaining 621 studies were reviewed further. Then 607 studies were excluded because they did not meet the eligible criteria or were redundant studies. Finally, fourteen studies were included (Figure 1) [13–26].

The fourteen studies were conducted in Italy, United Kingdom, Sweden, United States, Finland, Austria, and Canada, and were published between 1996 and 2015, and contained 9612 older patients with early breast cancer or DCIS. Of these fourteen studies, twelve studies evaluated the efficacy of radiotherapy in early breast cancer [13–15, 17–24, 26], and two studies evaluated the efficacy of radiotherapy in DCIS [16, 25]. Twelve studies were randomized clinical trials (RCT) [14–21, 23–26] and two studies were prospective cohort studies [13, 22]. In addition, at least 8439 patients (88.8%) were aged ≥ 60 years. The main characteristics of the included studies are summarized in Table 1.

**Table 1:** The main characteristics of included studies

Study	Country & year	Study type	Tumor type	Sample	Age	Follow up	Tumor characteristics	Intervention
Martelli	Italy 2015	Prospective cohort	EBC	627	≥ 70	17.4	T1N0M0:430/627(68.6%); T2N0M0:197/627(31.4%); ER+:542/627(86.4%); PR+:381/627(60.8%)	Tamoxifen + breast irradiation 50Gy/25F + boost 10Gy VS tamoxifen
Kunkler	UK 2015	RCT	EBC	1326	≥ 65; median:67	5	T1N0M0:1168/1326(88.1%); T2N0M0:158/1326(11.9%); ER+:1194/1326(90%); ER−:120/1326(9%); HR+:1326/1326(100%)	HT + breast irradiation 40-50Gy/15-25F + 16% boost 10-15Gy VS HT
Wickberg	Sweden 2014	RCT	EBC	199	≥ 55	20	T1N0M0:199/199(100%)	Breast irradiation 54Gy/27F + no boost VS no irradiation
Tinterri	Italy 2014	RCT	EBC	749	Range: 55-75; 411/749(54.9%) ≥ 65	9	T1:666/749(88.9%); T2:83/749(11.1%); N0:619/749(82.6%); N1:112/749(15%); ER+:692/749(92.4%); ER−:57/749(7.6%)	Breast irradiation 50Gy/25F + boost 10Gy VS no irradiation; HT: 652/749
Warnberg	Sweden 2014	RCT	DCIS	376	≥ 61	17.5	DCIS	Breast irradiation 50Gy/25F VS no irradiation
Hughes	USA 2013	RCT	EBC	636	≥ 70; 348/636(55%) ≥ 75	12.6	T1N0M0:623/636(98%); T2N0M0:13/636(2%); ER+:626/636(98.4%)	Tamoxifen + breast irradiation 45Gy/25F + boost 14Gy/7F VS tamoxifen
Williams	UK 2011	RCT	EBC	255	≥ 65	5	T0-2N0M0	Tamoxifen + breast irradiation 45-50Gy ± few boost 10-15Gy VS tamoxifen
Holli	Finland 2009	RCT	EBC	209	≥ 50	12.1	T1N0M0:209/209(100%); PR+:209/209(100%)	Breast irradiation 50Gy/25F + no boost VS no irradiation
Potter	Austria 2007	RCT	EBC	831	Mean:65.7(range:46-80) 817/831(98.3%) ≥ 50; 587/831(70.6%) ≥ 60; 293/831(35.3%) ≥ 70	4.48	T1N0M0:753/831(90.6%); T2N0M0:78/831(9.4%); HR+:831/831(100%); ER+:820/831(98.7%)	HT + breast irradiation 51 Gy±4Gy + 298/414 boost 10 ± 2Gy VS HT
Truong	Canada 2006	Prospective cohort	EBC	2438	≥65; 773/2438(31.7%) ≥ 75	7.5	T1:1956/2438(80.2%); T2:475/2438(19.5%); N0:1937/2438(79.5%); N1:501/2438(20.5%); ER+:1742/2438(71.5%)	Breast irradiation VS no irradiation; tamoxifen: ≥65: 1123/2438; ≥75: 377/773
Fyles	Canada 2004	RCT	EBC	769	≥50; median:68; 586/769(76.2%) ≥ 60; 325/769(42.3%) ≥ 70	5.6	T1N0M0:639/769(83.1%); T2N0M0:128/769(16.6%); HR+:621/769(80.7%)	Tamoxifen + breast irradiation 40Gy/16F + boost 12.5Gy/5F VS tamoxifen
Fisher	USA 2002	RCT	EBC	318	≥ 60; 100/318(%) ≥ 70	7.24	T1N0M0	Tamoxifen + breast irradiation 50Gy + 25%boost VS tamoxifen
Fisher	USA 1998	RCT	DCIS	294	≥ 60	7.5	DCIS	Breast irradiation 50Gy VS no irradiation
Forrest	UK 1996	RCT	EBC	585	≥ 60	5.7	T1:251/585(42.9%); T2:334/585(57.1%); N0:442/585(75.6%); N1:134/585(22.9%); ER+:343/585(58.6%)	Tamoxifen or chemotherapy + breast irradiation 50Gy/20-25F + no boost VS tamoxifen or chemotherapy

Abbreviations, DCIS: Ductal Carcinoma *In Situ*; EBC: Early Breast Cancer; ER: Estrogen Receptor; HR: Hormone Receptor; HT: Hormone Therapy; PR: Progestrone Receptor; RCT: Randomized Clinical Trial; UK: the United Kingdom; USA: the United States of America.

### Impact of radiotherapy on tumor relapse in early breast cancer

### Radiotherapy and local relapse

**Figure 2:**
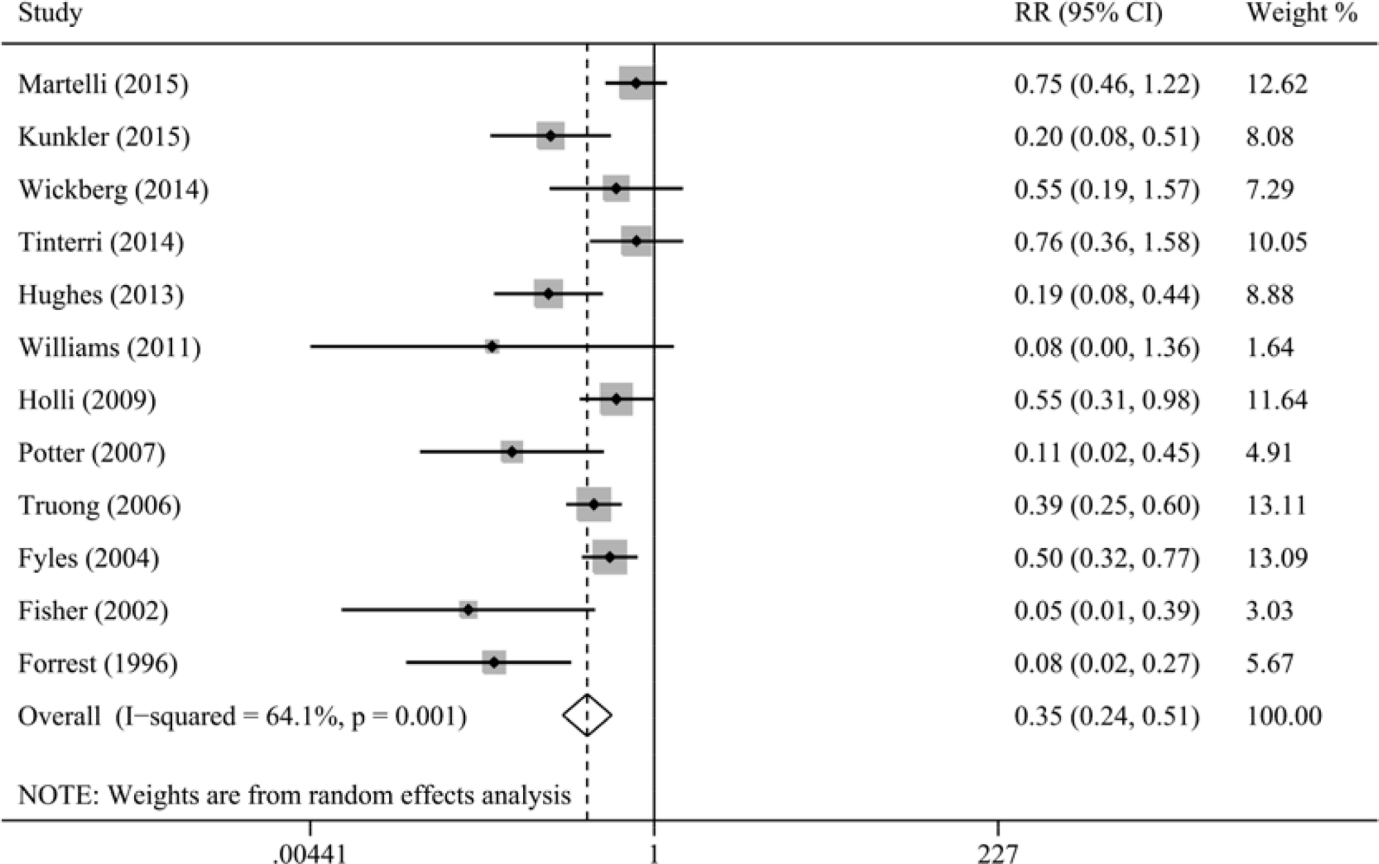
Meta-analysis of the association between radiotherapy and local relapse

Our results indicated that radiotherapy after BCS could reduce the risk of local relapse (HR = 0.25, 95% CI = 0.19–0.34, I^2^ = 41.3%; RR = 0.35, 95% CI = 0.24–0.51, I^2^ = 64.1%; Figure 2). Moreover, we specifically performed sensitivity analysis based on age (age ≥ 60 years: HR = 0.22, 95% CI = 0.15–0.33, I^2^ = 0.0%, RR = 0.22, 95% CI = 0.12–0.41, I^2^ = 66.4%; age ≥ 65 years: HR = 0.23, 95% CI = 0.15–0.35, I^2^ = 0.0%, RR = 0.31, 95% CI = 0.17–0.55, I^2^ = 58.7%; age ≥ 70 years: HR = 0.24, 95% CI = 0.13–0.43, I^2^ = 0.0%, RR = 0.37, 95% CI = 0.18–0.78, I^2^ = 67.9%). The sensitivity analyses based on study design, tumor characteristics, and tamoxifen obtained similar results, confirming the stability of our results.

Because AR and ARD were dependent on the duration of follow up, thus we assessed AR and ARD of local relapse based on median duration of follow up of approximately 5 and 10 years, respectively (group 1: 4.5–7.5 years and group 2: 8–17.5 years). The results of group 1 indicated that the AR of local relapse was 2.2% (101/4520; 95% CI = 1.8–2.7%) among radiotherapy group and 6.2% (175/2802; 95% CI = 5.3–7.1%) among non-radiotherapy group. The ARD was 4.0% (95% CI = 3.0–5.0%) and NNT was 25, in favor of radiotherapy, indicating approximately 25 patients were needed to be treated to prevent one case of local relapse. Sensitivity analysis based on age obtained similar results (age ≥ 65 years: AR = 2.0%, 95% CI = 1.5–2.5% for radiotherapy, AR = 4.9%, 95% CI = 3.9–5.9% for non-radiotherapy, ARD = 2.9%, 95% CI = 1.7–4.0%, NNT = 35; age ≥ 75 years: AR = 2.0%, 95% CI = 1.1–2.9% for radiotherapy, AR = 6.2%, 95% CI = 4.2–8.2% for non-radiotherapy, ARD = 4.2%, 95% CI = 2.0–6.4%, NNT = 24). For the group 2, the results indicated that the AR of local relapse was 5.3% (58/1091; 95% CI = 4.0–6.6%) and 10.5% (136/1299; 95% CI = 8.8–12.1%) among radiotherapy and non-radiotherapy group, respectively. And the ARD was 5.2% (95% CI = 3.0–7.3%) and NNT was 20, in favor of radiotherapy.

### Radiotherapy and IBTR

The results of IBTR indicated a lower risk of IBTR in patients who received radiotherapy compared with patients who did not receive radiotherapy (HR = 0.23, 95% CI = 0.09–0.60, I^2^ = 54.3%; RR = 0.25, 95% CI = 0.12–0.50, I^2^ = 67.8%). Similar results were obtained in the sensitivity analyses according to age, study design, tumor characteristics, and tamoxifen.

The results of group 1 indicated that the AR of IBTR was 0.6% (12/1892; 95% CI = 0.3–1.0%) and 5.0% (96/1906; 95% CI = 4.1–6.0%) among radiotherapy and non-radiotherapy group, respectively, with an ARD of 4.4% (95% CI = 3.4–5.4%) and NNT of 23, in favor of radiotherapy. In the group 2, the results indicated an AR of 3.0% (27/897; 95% CI = 1.9–4.1%) and 7.4% (83/1115; 95% CI = 5.9–9.0%) among radiotherapy and non-radiotherapy group, respectively. And the ARD was 4.4% (95% CI = 2.5–6.3%) and NNT was 23, in favor of radiotherapy.

### Radiotherapy and distant relapse

**Figure 3:**
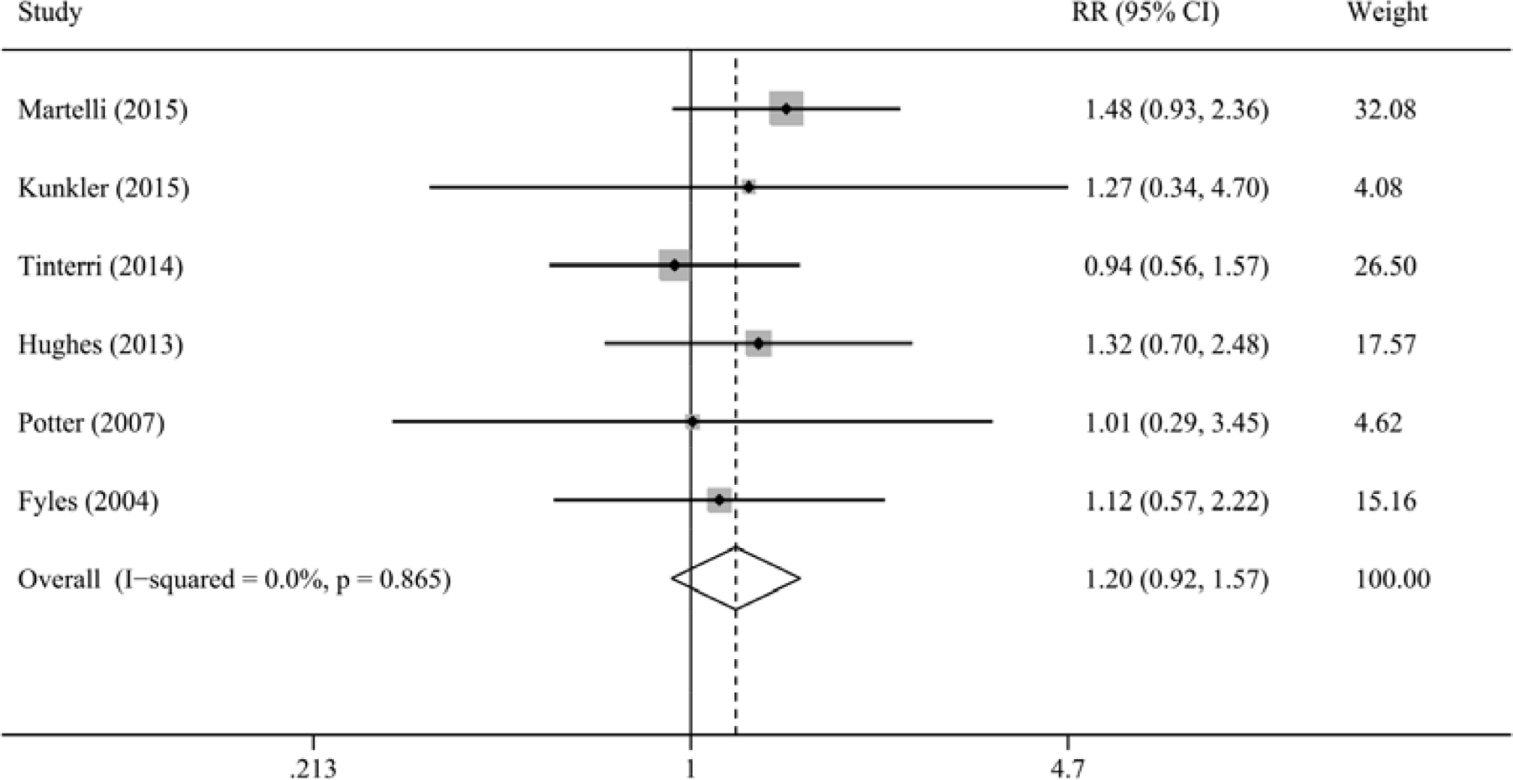
Meta-analysis of the association between radiotherapy and distant relapse

Our result indicated that radiotherapy after BCS could not reduce the risk of distant relapse (HR = 1.03, 95% CI = 0.77–1.39, I^2^ = 0.0%; RR = 1.20, 95% CI = 0.92–1.57, I^2^ = 0.0%; Figure 3). Moreover, we performed sensitivity analyses on the basis of favorable tumor characteristics, advanced age, RCT, and tamoxifen. The results still showed that the risk of distant relapse was not associated with radiotherapy after BCS.

Moreover, in the group 1, the pooled analyses for the AR and ARD indicated a similar risk of distant relapse between the radiotherapy group (AR = 2.1%; 45/2148; 95% CI = 1.5%-2.7%) and non-radiotherapy group (AR = 1.8%; 40/2163; 95% CI = 1.3%-2.4%) radiotherapy, and the ARD of distant relapse was insignificant (ARD = 0.2%, 95% CI = −0.6–1.1%). The results of group 2 obtained a similar tend (radiotherapy group: AR = 8.2%, 74/897, 95% CI = 6.4–10.1%; non-radiotherapy group: AR = 7.3%, 81/1115, 95% CI = 5.7–8.8%), with ARD of 1.0% (95% CI = −1.4–3.3%).

### Impact of radiotherapy on survival in early breast cancer

#### Radiotherapy and OS

**Figure 4:**
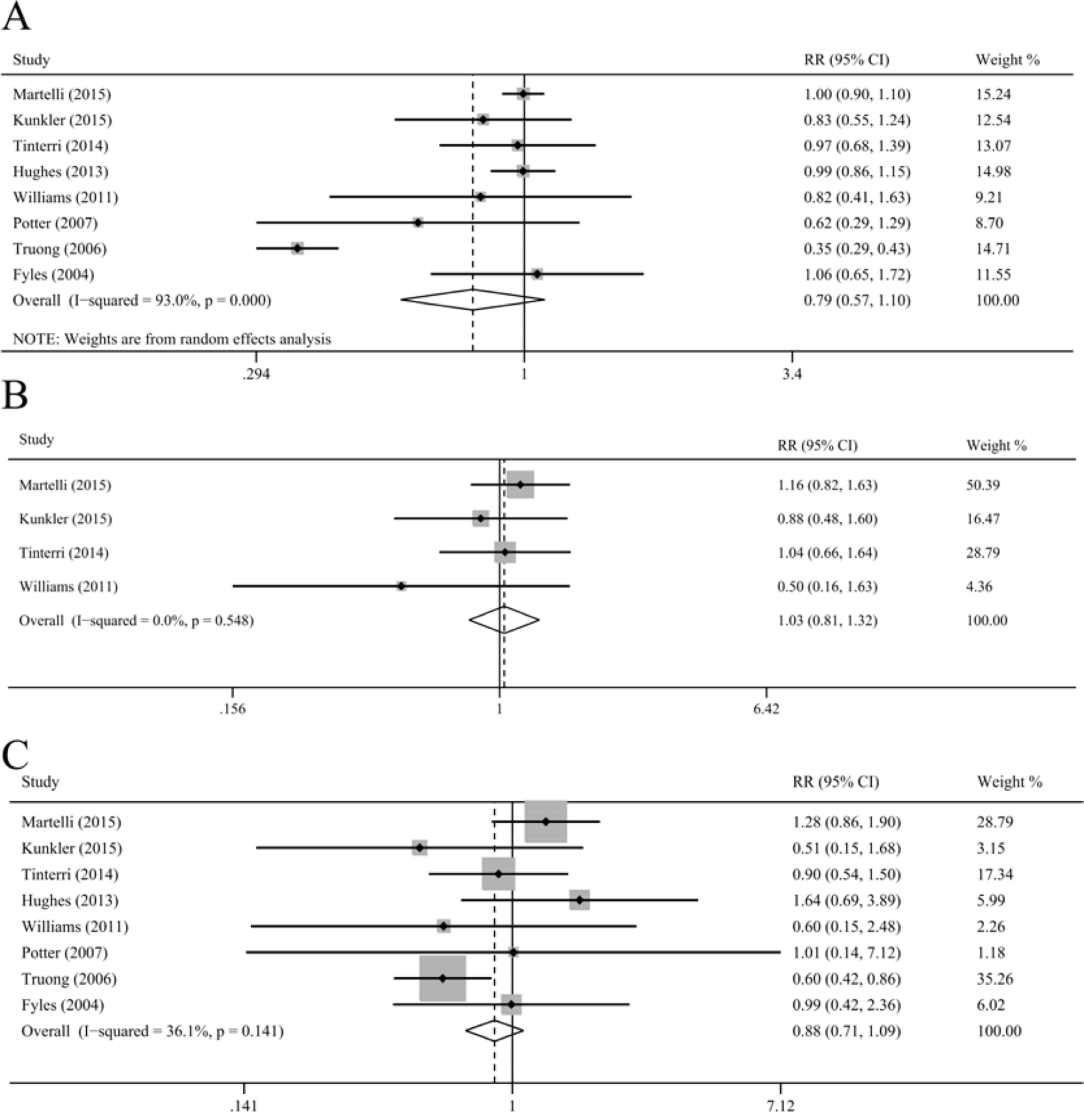
Meta-analysis of the association between radiotherapy and overall survival (**A**), cancer-specific survival (**B**), and breast-cancer-specific survival (**C**).

Our results indicated that OS did not differ significantly between the patients who received and did not receive radiotherapy (HR = 0.78, 95% CI = 0.59–1.04, I^2^ = 72.0%; RR = 0.79, 95% CI = 0.57–1.10, I^2^ = 93.0%; Figure 4), with substantial heterogeneity. Study by Truong et al. contributed substantial heterogeneity. Exclusion of the study by Truong et al. could increase statistical power and reduce heterogeneity, with similar results (HR = 0.92, 95% CI = 0.78–1.07, I^2^ = 0.0%; RR = 0.98, 95% CI = 0.91–1.06, I^2^ = 0.0%). Moreover, the sensitivity analyses stratified by age, study design, tumor characteristics, tamoxifen, and duration of follow up showed an insignificant association between radiotherapy and OS.

### Radiotherapy and CSS and breast-CSS and non-breast-CSS and non-CSS and other cancer-CSS

Radiotherapy after BCS could not improve the CSS, breast-CSS, non-breast-CSS, non-CSS, and other cancer-CSS in older patients with early breast cancer (CSS: RR = 1.03, 95% CI = 0.81–1.32, I^2^ = 0.0%; breast-CSS: HR = 0.80, 95% CI = 0.58–1.10, I^2^ = 44.6%, RR = 0.88, 95% CI = 0.71–1.09, I^2^ = 36.1%; non-breast-CSS: RR = 0.93, 95% CI = 0.82–1.05, I^2^ = 0.0%; non-CSS: RR = 0.94, 95% CI = 0.82–1.09, I^2^ = 0.0%; other cancer-CSS: RR = 1.03, 95% CI = 0.63–1.68, I^2^ = 0.0%; Figure 4). The sensitivity analyses based on age, study design, tumor characteristics, tamoxifen and follow up and the pooled analyses for the AR showed similar results.

### Radiotherapy and other secondary outcomes in early breast cancer

Our study also evaluated the associations between radiotherapy and subsequent mastectomy and contralateral breast cancer. Our results indicated that the risk of contralateral breast cancer was similar between the patients who received and did not receive radiotherapy (RR = 1.64, 95% CI = 0.84–3.23, I^2^ = 0.0%), with similar AR. In addition, radiotherapy reduced the frequency of subsequent mastectomy for local relapse in ipsilateral side (RR = 0.29, 95% CI = 0.12–0.73, I^2^ = 0.0%).

### Impact of radiotherapy in DCIS

There were only two RCT providing subgroup analyses comprising older patients with DCIS, with follow up of approximately 8 years [16, 25]. Radiotherapy could reduce the risk of ipsilateral breast events, including new DCIS or invasive cancer (radiotherapy group: AR = 9.2%, 95% CI = 6.3%-12.1%; non-radiotherapy group: AR = 26%, 95% CI = 21.8%-30.3%), with ARD of 16.8% (95% CI = 11.7–22.0%) and NNT of 6. However, our study could not obtain the results for the association between radiotherapy and survival, such as OS, CSS and breast-CSS, due to lack of data.

## DISCUSSION

Postoperative radiotherapy is an integral part of treatment to reduce the local relapse in patients with early breast cancer [4]. However, the treatment recommendations for early breast cancer are mainly based on clinical studies that frequently include young and exclude older patients, and the use of radiotherapy after BCS obviously declines with advancing age [10]. Thus, there are no consistent agreements about whether radiotherapy could provide substantial local control and survival benefit for older patients with early breast cancer. Our meta-analysis of 14 studies provides evidence of an association between radiotherapy use and high local control in older patients with early breast cancer. However, radiotherapy use cannot translate into significant survival benefits.

Our study indicated that radiotherapy after BCS could reduce the risk of local relapse in older patients with early breast cancer. The AR of local relapse was 2.2% and 6.2% for patients who received and did not receive radiotherapy after approximately 5 years, respectively, corresponding with ARD of 4.0% and NNT of 25. Besides, the ARD for local relapse was 5.2% after a longer duration of follow up of approximately 10 years. However, radiotherapy could not improve survival benefits, including OS, CSS, breast-CSS, non-breast-CSS, non-CSS and other cancer-CSS. The results were confirmed by in-depth sensitivity analyses. Moreover, radiotherapy could reduce the risk of ipsilateral breast events in older patients with DCIS.

Clinically, age is an important and necessary factor to be considered in the use of radiotherapy in patients with early breast cancer. Compared with younger patients, older patients are more likely to have higher comorbidity, worse tolerability for treatment, and shorter life expectancy, which may have a negative influence on survival benefits. Indeed, clinical treatments for older patients with early breast cancer were often less aggressive, and epidemiological studies also reported that there was significant reduction in use of radiotherapy with increasing age for patients with early breast cancer [9, 10]. Moreover, the EBCTCG trial showed that the risk of local relapse fell with increasing age in both radiotherapy and no radiotherapy group for early breast cancer after BCS, as did the ARD [27, 28]. The NSABP B-21 trial also showed that the risk of IBTR was reduced with advancing age in early breast cancer [24]. Liljegren et al. showed that risk for local relapse decreased by 3% (95% CI, 1% to 6%) per year of increasing age [29]. The results of the current study indicated that the 5-year and 10-year ARD for local relapse was 4.0% and 5.2%, respectively, and the 5-year and 10-year ARD for IBTR was 4.4% and 4.4%, respectively. Although our results indicated that radiotherapy after BCS could slightly reduce the risk of local relapse and IBRT, the absolute benefits from radiotherapy may be less because ARD was low, resulting in a high NNT for preventing one local relapse. Besides, our results showed that there were no differences for the survival. Similarly, the PRIME, PRIME II, and CALGB 9343 trials reported that radiotherapy could only improve local control but not survival [14, 18, 19].

As mentioned, adding radiotherapy to BCS did not translate into significant survival benefits in older patients with early breast cancer. It is probably that a survival benefit was difficult to be detected in older patients with early breast cancer treated with radiotherapy because they had good survival even without radiotherapy. Moreover, several studies have reported that breast cancer tend to behave more indolently in older patients, and the postmenopausal status was related to favorable survival [7, 8]. And tamoxifen could also contribute to a substantial reduction in the risk of tumor relapse and thus weaken the benefits from radiotherapy [27, 30]. Indeed, Fyles et al. included postmenopausal patients with tamoxifen administration and reported that the rate of 5-year OS was 93.2% and only 10 deaths were due to breast cancer in non-radiotherapy group [23]. CALGB 9343 trial showed that the OS, in non-radiotherapy group, was 86% and only 3 deaths were due to breast cancer after 5-year follow up, and 10-year OS was 66% and 10-year breast-CSS was 98% after 12.6-year follow up [18, 31]. Furthermore, radiotherapy may increase mortality from heart disease and lung cancer, resulting in counteracting the slight benefit for breast cancer [32, 33]. Thus, individual and tailored strategies on indication of radiotherapy were important and should be precisely explored.

Clinically, it was important to identify subpopulation who could safely and effectively omit the radiotherapy after BCS. The main concern was the definition of older age for patients with early breast cancer. However, there was no consistent definition of older age and the definition of older age varied from 50 to 70 in included studies. In our study, the “older” not only focused on the objective age but also on the menopausal status because postmenopausal status maybe reflect physiological status for “older” and was a favorable survival factor. Our study included at least 88.8% patients were aged ≥ 60. Furthermore, sensitivity analysis based on age obtained similar results. Unfortunately, our study could not determine the optimal definition of older age due to a lack of data. Future studies are required to establish an optimal cut-off value for older age, considering the fact that a higher cut-off value may miss a substantial number of patients in clinical practice. In addition, the tumor and patient characteristics were also important factors for the selection of specific subpopulation. The majority of patients in our study had relatively favorable tumor and patient characteristics: T1, negative lymph node, positive hormone receptor, postmenopausal status, and tamoxifen treatment. Several studies also showed that grade-3 tumor and lymphovascular invasion may be an important factor for the use of radiotherapy [13, 27, 34]. Whether the definition of favorable tumor characteristics should be based on additional biomarker (i.e., Ki-67, cyclooxygenase-2, and E-cadherin) in early breast cancer was unclear.

In clinical practice, it was important to judge whether the benefit of radiotherapy outweighed its adverse effects. Older patient frequently had worse performance status and higher comorbidity, and radiotherapy-related toxicities and complications may frequently occur [32, 33], and the improvement of local control by radiotherapy could not translate into survival. Moreover, radiotherapy could result in inconvenience, worse cosmetic results and adverse effects, such as breast pain, breast edema, and skin-color changes, reducing quality of life [31]. Recently, several studies have showed that accelerated partial breast radiotherapy or intraoperative radiotherapy may become safe, effective and alternative strategies with acceptable toxicities and short time durations [35, 36].

The main strength of the current meta-analysis study was that this meta-analysis systematically evaluated the efficacy of radiotherapy in early breast cancer and DCIS through assessing all the relevant end points and included all eligible studies to increase statistical power though increasing the number of patients. However, there were several limitations in the current study. First, this study was based on published data and we could not obtain detailed individual data. Future high-quality, well-designed multicenter studies are required. Second, although the two studies on DCIS showed that radiotherapy could not improve survival for DCIS patients in overall group, irrespective of age, we could not specifically evaluate the survival benefit of radiotherapy in older patients due to lack of data and study number. Future studies are needed to explore the impact of radiotherapy on survival in older patients with DCIS after BCS. Third, our study did not provide a conclusive result regarding the optimal definition of older age. Fourth, although our results indicated that radiotherapy after BCS could slightly reduce the risk of local relapse, there were no studies performing cost-analysis to evaluate whether radiotherapy was a cost-effective treatment for local relapse. Thus, further studies are needed to evaluate its cost-effectiveness.

In conclusion, although our study indicates that radiotherapy after BCS could slightly reduce the risk of local relapse in older patients with favorable early breast cancer, radiotherapy can only gain low absolute benefits and high NNT for local relapse. Moreover, radiotherapy cannot translate into significant survival benefits. Thus, our study proposes that radiotherapy options should not only focus on the relative risk of local relapse but also on the absolute benefit, NNT and survival benefits.

## MATERIALS AND METHODS

### Literature search

PubMed and Embase database were systematically searched for relevant studies evaluating the efficacy of postoperative radiotherapy after BCS in older patients with early breast cancer or DCIS (to August 2016). The main search terms were “breast-conserving surgery”, “breast-conservation surgery”, “lumpectomy”, “local excision mastectomy”, “segmental mastectomy”, “limited resection mastectomy”, “radiotherapy”, “irradiation therapy”, “radiation therapy”, “chemoradiotherapy”, “breast cancer”, “older”, “elderly, “old”, “geriatrics”, “early stage”, “T1-T2”, “stage I-IIA”, and “*in situ*”. The reference lists of reviews and relevant studies were also checked for potential studies.

### Eligibility criteria

Studies were included if they met all of the following criteria: (1) assessment of the efficacy of postoperative radiotherapy after BCS in older patients with early breast cancer or DCIS; (2) the definition of older patients was postmenopausal patient aged ≥ 50 year or patient aged ≥ 55 year regardless of postmenopausal status; (3) the study design was prospective study with at least a subgroup analysis containing older patients; (4) the outcomes of interest were tumor relapse (local relapse, distant relapse, ipsilateral breast tumor relapse [IBTR], and contralateral breast cancer) or/and survival (overall survival [OS], cancer-specific survival [CSS], breast cancer-specific survival [breast-CSS], non-breast cancer-specific survival [non-breast-CSS], non-cancer-specific survival [non-CSS], and other cancer-specific survival [other cancer-CSS]); and (5) the effect estimates and corresponding 95% confidence intervals (CIs) could be extracted directly or calculated from published data indirectly. To retain maximum information, the most recent study was included and the data that only reported in excluded duplicated studies were extracted and added it into the included duplicated study if there were several studies based on the same population or data set.

### Data extraction

Studies were reviewed and data were extracted by two reviewers, independently. For each study, the following information was extracted: first author, publication year and country, study design, sample size, patient age, follow-up duration, treatment strategy in experimental and control group, tumor characteristics, and outcome of study. IBTR was defined as any cancer in ipsilateral breast and local relapse was defined as IBTR or any relapse in ipsilateral regional lymph nodes. And second cancer in the ipsilateral breast was included as an event for local relapse. Any disagreements on the data extraction were resolved through discussion.

### Statistical analysis

Hazard ratios (HRs) and risk ratios (RRs) were used as effect measures to evaluate the efficacy of postoperative radiotherapy after BCS in older patients with early breast cancer or DCIS. The HRs and 95% CIs were calculated from the published data using the method designed by Tierney if the values were not provided directly [37]. In addition, absolute risk (AR) and absolute risk difference (ARD) was calculated to evaluate absolute benefit of radiotherapy. The AR and ARD were calculated based on the duration of follow up because AR and ARD were dependent on the duration of follow up. The number needed to treat (NNT) was calculated as 1 divided by ARD, and NNT was the number of patients who need to be treated to expect there to be one additional event to be observed.

An overall analysis was performed by enrolling all the eligible studies. To evaluate the impact of age on the efficacy of radiotherapy, a sensitivity analysis was performed based on age. Besides, sensitivity analyses were also performed based on tumor characteristics, study design, and tamoxifen. Cochran Q test and the *I*^2^ statistic were used to evaluate heterogeneity [38]. A random-effects model was used if significant heterogeneity existed; otherwise, the fixed-effects model was used. Begg's and Egger's tests was used to evaluate publication bias, and trim-and-fill analysis was performed to assess the effect of publication bias [39–41].

All statistical analyses were performed using Stata software 12.0 (Stata Corporation, College Station, TX, USA). A two-sided *P* value < 0.05 was considered statistically significant.

## Abbreviations

AR: Absolute Risk
ARD: Absolute Risk Difference
BCS: Breast-Conserving Surgery
CALGB: Cancer and Leukemia Group B
CI: Confidence Intervals
CSS: Cancer-Specific Survival
DCIS: Ductal Carcinoma *In Situ*
EBCTCG: the Early Breast Cancer Trialists’ Collaborative Group
HR: Hazard Ratio
IBTR: Ipsilateral Breast Tumor Relapse
NNT: Number Needed to Treat
NSABP: the National Surgical Adjuvant Breast and Bowel Project
OS: Overall Survival
PRIME: the Post-operative Radiotherapy In Minimum-risk Elderly
RCT: Randomized Clinical Trial
RR: Risk Ratio
